# Bionic sleep therapy combined with multimodal management for chronic insomnia with alcohol withdrawal-related symptoms: A case report

**DOI:** 10.1097/MD.0000000000050991

**Published:** 2026-10-09

**Authors:** Shuai-Yu Zhu, Qiu-Xia Xiao, Zheng-Yuan Duan, Yan-Yan Ma, Peng-Cheng Zhao, Hong-Xia Chen, Yi-Jun Bao, Jiang-Ming Lei, Lin Wang, Huan Zhou, Run-Xing Ma, Hui-Li Pu, Kai-Feng Wu, Liu-Lin Xiong, Ruo-Lan Du

**Affiliations:** a Department of Anesthesiology, Sleep Therapy Clinical Medicine Center, Brain-Computer Interface Medical Center, The Third Affiliated Hospital of Zunyi Medical University (The First People’s Hospital of Zunyi), Zunyi, Guizhou, China; b Department of Laboratory Medicine/Scientific Research Center, The Third Affiliated Hospital of Zunyi Medical University (The First People’s Hospital of Zunyi), Zunyi, Guizhou, China.

**Keywords:** alcohol withdrawal, bionic sleep therapy, dexmedetomidine, insomnia, propofol

## Abstract

**Rationale:**

Patients who use alcohol as a sleep aid may experience withdrawal-related insomnia and hyperarousal, which can reinforce continued drinking. Bionic sleep therapy (BST) is an exploratory intervention using intravenous anesthetic and sedative agents under close monitoring to induce a brief, controllable, sleep-like state. It may provide short-term support for sleep initiation during early alcohol withdrawal.

**Patient concerns:**

A 71-year-old man had chronic insomnia for over 15 years and consumed 100 to 200 mL of Chinese baijiu nightly to initiate sleep, with additional alcohol after nocturnal awakenings. Repeated attempts to stop drinking were followed by worsening insomnia, restlessness, palpitations, diaphoresis, and intense alcohol craving, which improved after drinking.

**Diagnoses:**

Chronic insomnia with recurrent mild alcohol withdrawal-related symptoms and a high risk of complicated alcohol withdrawal.

**Interventions:**

The patient underwent 2 monitored BST sessions on hospital days 1 and 2 under continuous cardiorespiratory monitoring. Propofol (1 mg/kg) was administered intravenously for rapid induction, followed by a 60-minute dexmedetomidine infusion via an IV pump at 0.2 to 0.4 μg/kg/h. Multimodal treatment was intensified on hospital day 2, including cognitive behavioral therapy for insomnia, medication adjustment, and adjunctive neuromodulation.

**Outcomes:**

During the overall multimodal treatment course, on the night of the first BST session, the patient initiated sleep without alcohol and returned to sleep after a nocturnal awakening without further drinking. Polysomnography showed a total sleep time of 373 minutes and sleep efficiency of 85.6%. Following the second session, he consumed approximately 20 mL of alcohol after a nocturnal awakening before returning to sleep; sleep diary-reported sleep efficiency was 90.6%. Vital signs remained stable during both sessions, and no apparent anesthesia- or sedation-related adverse events occurred. No further alcohol intake was recorded from hospital day 3 to day 7. At discharge, subjective sleep efficiency was 87.7%, with improvements in sleep initiation, maintenance, and some daytime symptoms. Postdischarge follow-up data were unavailable.

**Lessons:**

BST using propofol and dexmedetomidine may warrant further investigation as an adjunct to multimodal management in patients with chronic insomnia, long-term alcohol use as a sleep aid, and withdrawal-related symptoms. However, improvements cannot be attributed specifically to BST, and its contribution and durability require prospective evaluation.

## 1. Introduction

Insomnia is a prevalent sleep disorder characterized by difficulty initiating or maintaining sleep, early-morning awakening, and associated daytime impairment.^[1,2]^ Some patients use alcohol to alleviate insomnia symptoms. Although the acute sedative effects of alcohol may transiently facilitate sleep onset, long-term use disrupts sleep homeostasis and architecture and may progressively lead to tolerance and dependence. Reducing or discontinuing alcohol intake can precipitate withdrawal-related insomnia, anxiety, irritability, and craving, increasing the likelihood of relapse and perpetuating a vicious cycle of “insomnia—alcohol use for sleep—withdrawal-related insomnia—resumption of drinking.”^[3,4]^

Cognitive behavioral therapy for insomnia (CBT-I) is a first-line treatment for chronic insomnia that improves insomnia-related cognitions, behaviors, and long-term outcomes; however, it typically requires a sustained treatment period and consistent patient engagement.^[5,6]^ Alcohol withdrawal management primarily focuses on risk assessment, symptom control, and prevention of severe complications. Benzodiazepines can rapidly alleviate withdrawal symptoms but do not directly address the entrenched behavioral pattern of alcohol use as a sleep aid and may be associated with oversedation, respiratory depression, and dependence.^[7,8]^ Thus, for clinically stable patients who continue to experience marked difficulty initiating sleep after reducing or discontinuing alcohol, strategies that provide rapid, short-term sleep support within standard withdrawal management warrant further investigation.

Bionic sleep therapy (BST) is an exploratory monitored pharmacologic intervention recently described in a preliminary clinical report from China,^[9]^ in which intravenous anesthetic and sedative agents are administered under continuous cardiorespiratory and sleep monitoring to induce and maintain a brief, controllable, sleep-like sedative state.

The intervention aims to reduce insomnia-related hyperarousal, facilitate sleep initiation, and create a therapeutic window for subsequent sleep consolidation and comprehensive treatment.^[9]^ Potential advantages include rapid onset, controllability, and a smooth transition to subsequent therapy. For patients with long-term reliance on alcohol for sleep who develop withdrawal-related insomnia and pronounced difficulty initiating sleep after reducing or discontinuing alcohol, BST may facilitate alcohol-free sleep initiation and provide short-term support during early withdrawal and transition to multimodal treatment. Distinct from the previously reported use of BST for refractory insomnia, the present case involved chronic insomnia complicated by long-term alcohol use specifically for sleep and recurrent withdrawal-related symptoms during cessation attempts. We report a case of an older patient with long-term alcohol use as a sleep aid and withdrawal-related symptoms who received BST combined with multimodal management, and describe short-term in-hospital changes in sleep, withdrawal-related symptoms, and alcohol use (Fig. 1).

**Figure 1.**
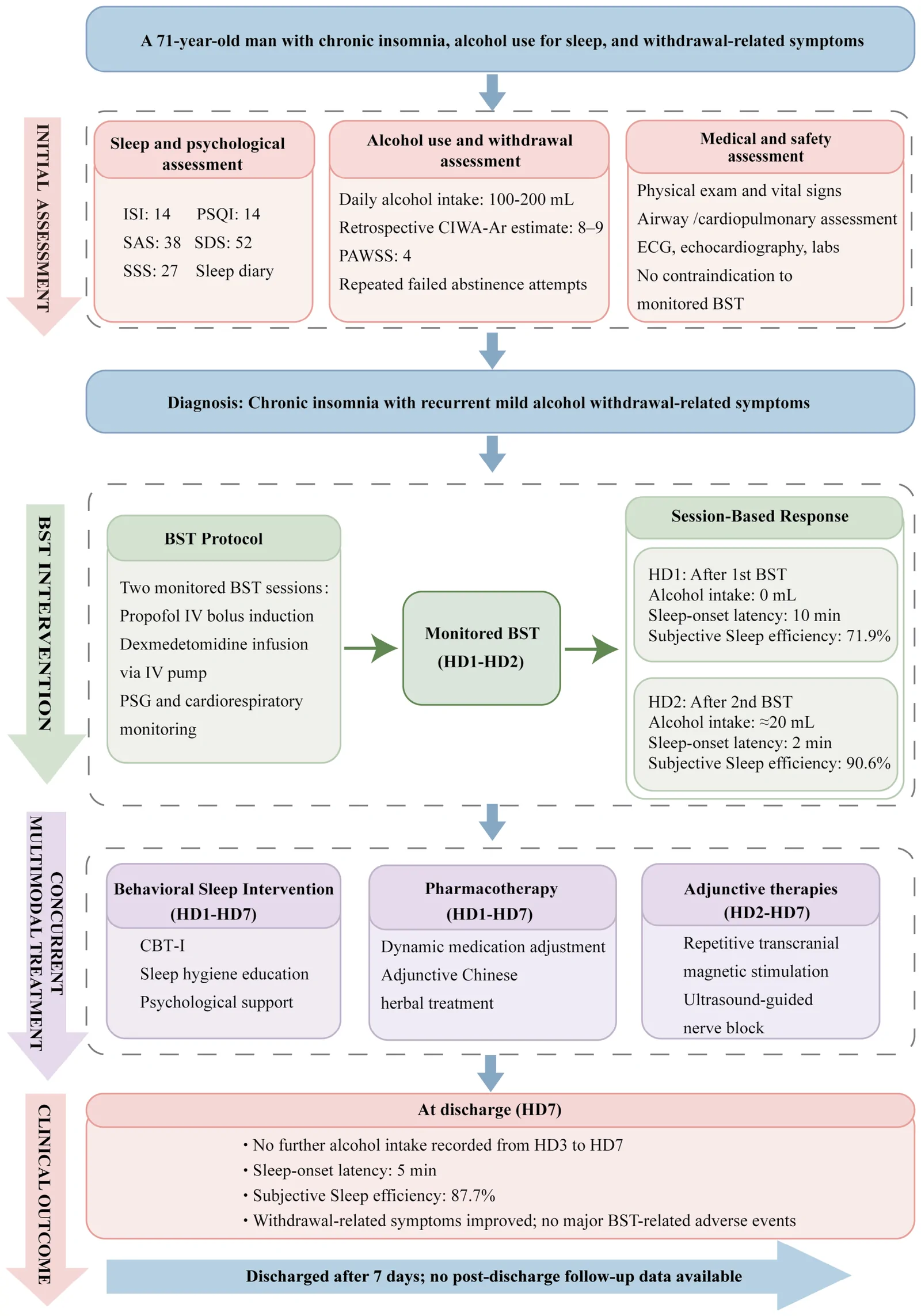
Clinical timeline of patient assessment, monitored bionic sleep therapy, multimodal treatment, and in-hospital outcomes. Sleep-onset latency and subjective sleep efficiency shown in the Session-Based Response panel were derived from the patient’s sleep diary. BST = bionic sleep therapy, CBT-I = cognitive behavioral therapy for insomnia, CIWA-Ar = Clinical Institute Withdrawal Assessment for Alcohol, Revised, ECG = electrocardiography, HD = hospital day, ISI = Insomnia Severity Index, IV = intravenous, PAWSS = Prediction of Alcohol Withdrawal Severity Scale, PSG = polysomnography, PSQI = Pittsburgh Sleep Quality Index, SAS = Self-Rating Anxiety Scale, SDS = Self-Rating Depression Scale, SSS = Somatic Self-Rating Scale.

## 2. Case presentation

A 71-year-old man (height 168 cm; weight 57.0 kg; body mass index 20.19 kg/m^2^) presented to our sleep medicine center with a >15-year history of poor sleep that had worsened over the preceding 3 years. Approximately 15 years earlier, he had begun using alcohol as his primary sleep aid, consuming 100 to 200 mL of Chinese baijiu after dinner each evening. He typically drank to the point of mild, and at times overt, intoxication before falling asleep, and often drank again after nocturnal awakenings to return to sleep. Over the preceding 3 years, the amount of alcohol consumed both before sleep and after awakenings progressively increased to achieve a similar sleep-promoting effect. Concurrently, his sleep became lighter and more fragmented, with more frequent awakenings and increasing difficulty maintaining sleep. He also developed reduced daytime energy, subjective memory decline, diminished interest, and low mood.

The patient had made several attempts to stop drinking on his own; however, within hours of each attempt, he developed irritability, restlessness, palpitations, diaphoresis, worsening difficulty initiating sleep, and intense alcohol craving. Some episodes were also accompanied by headache and loss of appetite. These symptoms typically intensified late at night and were relieved only after resuming drinking. Repeated failed attempts reinforced his entrenched belief that “I simply cannot sleep without alcohol.” Although he had previously tried sleep medications, including Limian Ning capsules and estazolam tablets, he continued to rely on alcohol to initiate sleep and eventually abandoned further attempts to stop drinking on his own. As his alcohol intake progressively increased, his sleep became increasingly shallow and fragmented. Given his long-standing difficulty initiating sleep, impaired sleep maintenance, and repeated failed cessation attempts, he was admitted for further evaluation and treatment.

The patient continued to consume alcohol immediately before admission. On admission, he was alert and fully oriented. No tremor, perceptual disturbances, seizures, or delirium were observed, although diaphoresis and restlessness were present. He had a >10-year history of hypertension and was receiving regular medical treatment; his admission blood pressure was 123/81 mm Hg. He denied a history of coronary artery disease, epilepsy, or traumatic brain injury. A physical examination revealed mandibular retrognathia and an enlarged tongue, without obvious narrowing of the pharyngeal airway. Echocardiography showed mild aortic and pulmonic regurgitation with normal left ventricular systolic function. The remainder of the routine physical examination and laboratory investigations revealed no clinically significant abnormalities.

After admission, the patient underwent systematic sleep, psychological, and physical assessments. His Pittsburgh Sleep Quality Index score was 14, indicating impaired sleep quality. His Insomnia Severity Index score was 14, within the subthreshold insomnia range. Self-Rating Anxiety Scale and Self-Rating Depression Scale scores were 38 and 52, respectively, both below positive cutoff values. His Somatic Self-Rating Scale score was 27, within the normal range. Heart rate variability-based stress assessment showed a physical stress index of −0.87, a psychological stress index of −0.56, and a stress-resistance score of 104.9, suggesting no marked elevation in physical or psychological stress at the time of testing.

Based on the medical history, physical examination, and assessments, the clinical team further evaluated the patient’s prior alcohol withdrawal-related episodes. During multiple previous cessation attempts, he developed insomnia, anxiety, restlessness, palpitations, and diaphoresis within hours of alcohol cessation, showing a clear temporal relationship with stopping alcohol use; these symptoms improved after resuming drinking. He had no history of withdrawal-related seizures, hallucinations, delirium, or impaired consciousness. These features were consistent with the clinical features of alcohol withdrawal described in the Diagnostic and Statistical Manual of Mental Disorders, Fifth Edition.^[10]^ Based on the patient’s recollection of typical previous episodes, the Clinical Institute Withdrawal Assessment for Alcohol, Revised, items were retrospectively estimated, yielding a total score of approximately 8 to 9, consistent with a mild range of withdrawal severity. His Prediction of Alcohol Withdrawal Severity Scale score was 4, meeting the screening threshold for a high risk of complicated alcohol withdrawal. Overall, the patient was diagnosed with chronic insomnia accompanied by a history of recurrent mild alcohol withdrawal-related symptoms and a high risk of complicated alcohol withdrawal.

On hospital day 1, before the first BST session, the treatment team advised the patient to stop drinking, administered alprazolam for insomnia, and initiated sleep hygiene education. Given his long-standing use of alcohol to cope with difficulty initiating sleep and nocturnal awakenings, together with multiple unsuccessful cessation attempts before admission, BST was scheduled for the first night of hospitalization after assessment of the risk of complicated alcohol withdrawal, anesthetic safety evaluation, and multidisciplinary discussion. The intervention was intended to help the patient initiate and maintain sleep without alcohol under close monitoring, providing a short-term transitional window for subsequent support for alcohol cessation, behavioral sleep intervention, and comprehensive treatment.

The first BST session began at 22:30 on the night of admission. In the sleep laboratory, the patient underwent polysomnography (PSG) with continuous electrocardiographic and respiratory monitoring. Peripheral intravenous access was established, and supplemental oxygen was administered. At 22:50, propofol 1 mg/kg was given as an intravenous bolus to induce rapid sedation, followed by a continuous dexmedetomidine infusion via an IV pump at 0.2 to 0.4 μg/kg/h for 60 minutes to maintain a sleep-like state. Vital signs remained stable throughout the session. The patient consumed no alcohol that night and fell asleep successfully under monitoring. He experienced 1 nocturnal awakening but returned to sleep without further alcohol intake and slept until the following morning (Fig. 2A and B). On awakening, he was alert and fully oriented and reported a better sleep experience than before admission. He did not experience the irritability, anxiety, palpitations, or diaphoresis that had typically occurred during previous cessation attempts. Under this pharmacologically induced condition, PSG showed a total sleep time of 373 minutes and a sleep efficiency of 85.6%; N3 sleep was absent, and rapid eye movement sleep accounted for 7.5% of total sleep time (Fig. 2A). The sleep diary indicated a self-reported sleep-onset latency of 10 minutes, subjective sleep efficiency of 71.9% (Fig. 2C and D), a sleep quality score of 4, and a next-day alertness score of 3 (Fig. 2E).

**Figure 2.**
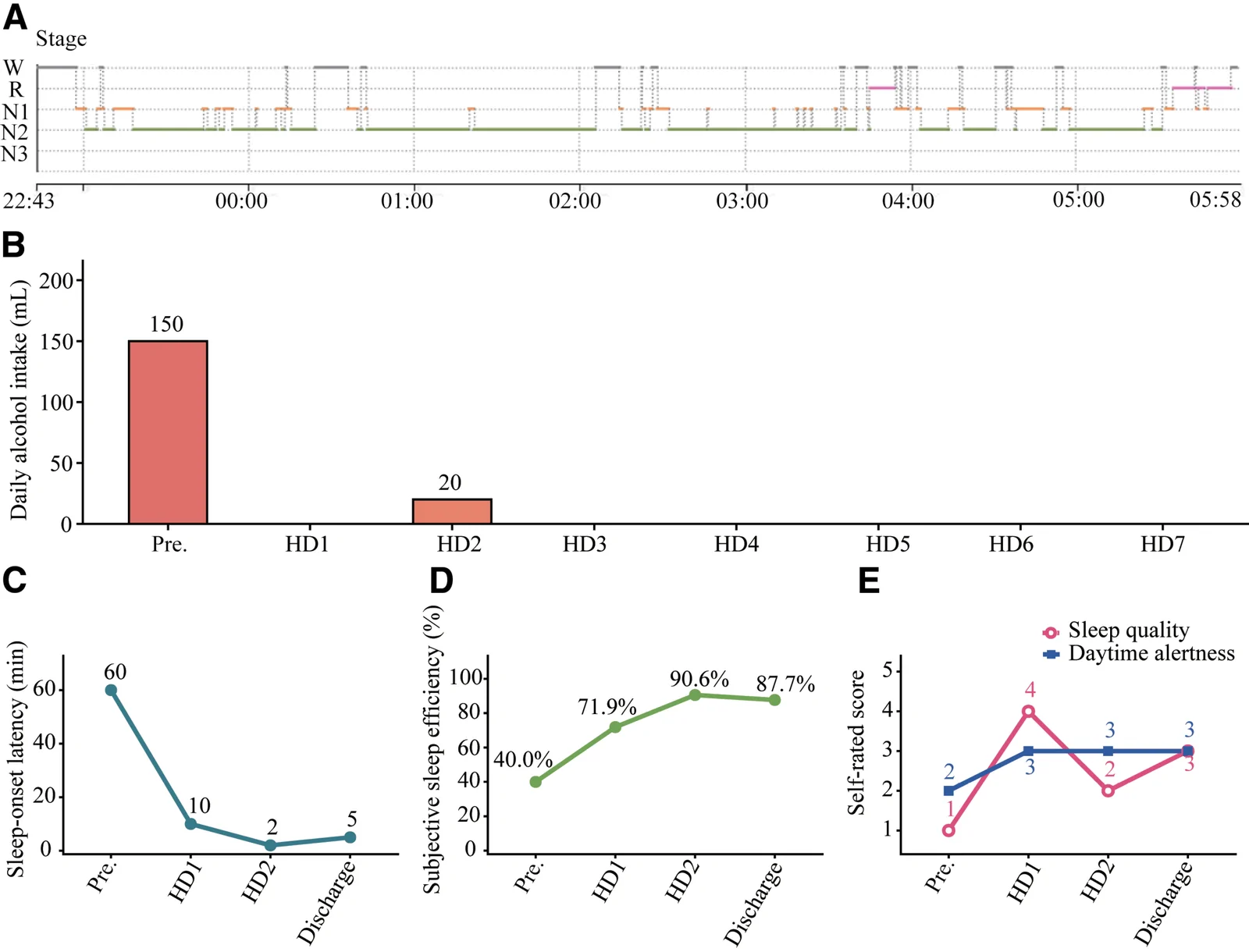
Changes in alcohol intake and sleep-related measures during hospitalization. (A) Polysomnographic hypnogram from the night of admission, during which the first BST session was performed. Gray, magenta, orange, and green segments indicate wake (W), rapid eye movement sleep (R), N1 sleep, and N2 sleep, respectively. (B) Daily alcohol intake before admission and during hospitalization. (C) Sleep diary-reported sleep-onset latency before admission, after the first BST session on hospital day 1, after the second BST session on hospital day 2, and at discharge. (D) Sleep diary-reported subjective sleep efficiency before admission, after the first BST session on hospital day 1, after the second BST session on hospital day 2, and at discharge. (E) Patient-reported sleep quality and daytime alertness before admission, after the first BST session on hospital day 1, after the second BST session on hospital day 2, and at discharge. Both sleep quality and daytime alertness were rated on a 5-point scale (1–5), with higher scores indicating better sleep quality and greater daytime alertness. BST = bionic sleep therapy, HD = hospital day, N1, N2, and N3 = stages 1, 2, and 3 of non-rapid eye movement sleep, Pre. = pre-admission, R = rapid eye movement sleep, W = wakefulness.

After the first BST session, the patient showed greater acceptance of and confidence in subsequent treatment. On hospital day 2, the treatment team further intensified multimodal management. Sleep hygiene education was reinforced, and CBT-I-based intervention was provided together with psychological support. Pharmacotherapy was adjusted according to his sleep and emotional status and included bedtime alprazolam and eszopiclone, with the addition of Shugan Jieyu capsules. The patient also received adjunctive repetitive transcranial magnetic stimulation and ultrasound-guided nerve block to improve sleep-related symptoms and autonomic regulation. That evening, he underwent a second BST session under continuous cardiorespiratory monitoring. The treatment protocol was the same as that used for the first session, and a sleep-like state was rapidly induced.

On the night of the second BST session, the patient independently consumed approximately 20 mL of baijiu after a nocturnal awakening and subsequently returned to sleep. The concurrent sleep diary showed a subjective sleep-onset latency of 2 minutes, subjective sleep efficiency of 90.6%, sleep quality score of 2, and next-day alertness score of 3. After further discussion and psychological support, the patient demonstrated limited motivation to abstain from alcohol and expressed difficulty changing his long-established habit of using alcohol as a sleep aid. He therefore declined a third BST session. Vital signs remained stable during both BST sessions, and no apparent anesthesia- or sedation-related adverse events were observed.

From hospital days 3 to 7, after the patient declined a third BST session, the treatment team continued sleep hygiene education, CBT-I, physical modalities, and traditional Chinese medicine-based treatment. Pharmacotherapy was adjusted according to his sleep and emotional status and included intranasal dexmedetomidine, lorazepam, mirtazapine, and escitalopram. During this period, the patient became more accepting of and confident in attempting to sleep without alcohol. No further alcohol intake was recorded during the later phase of hospitalization (hospital days 3–7). The patient subjectively reported a shorter sleep-onset latency than before admission, fewer nocturnal awakenings, and an improved ability to return to sleep after awakening. He also reported improvement in daytime symptoms, including dizziness, headache, fatigue, and irritability. No further prominent withdrawal-related manifestations, such as diaphoresis or psychomotor agitation, were recorded. Once his clinical condition was relatively stable, the patient requested discharge. At discharge, his sleep diary showed a subjective sleep efficiency of 87.7%. Because he did not return for the scheduled postdischarge visit, no postdischarge follow-up data were obtained.

## 3. Discussion

This case describes an older patient with long-standing reliance on alcohol as a sleep aid and withdrawal-related insomnia who received BST, using propofol for induction and dexmedetomidine for maintenance, combined with multimodal management including sleep hygiene education, CBT-I, psychological support, and medication adjustment. On the night of the first BST session, the patient was able to initiate sleep without alcohol and return to sleep after a nocturnal awakening without further drinking. No further alcohol intake was recorded from hospital days 3 to 7, and improvements were also reported in sleep maintenance and several daytime symptoms. These observations suggest that BST may provide early transitional support within a comprehensive treatment strategy by facilitating initial alcohol-free sleep and creating a therapeutic window for subsequent multimodal management. More broadly, this case provides preliminary clinical support for further exploration of BST as part of an integrated treatment approach for complex presentations involving chronic insomnia, alcohol use as a sleep aid, and withdrawal-related symptoms (Fig. 3).

**Figure 3.**
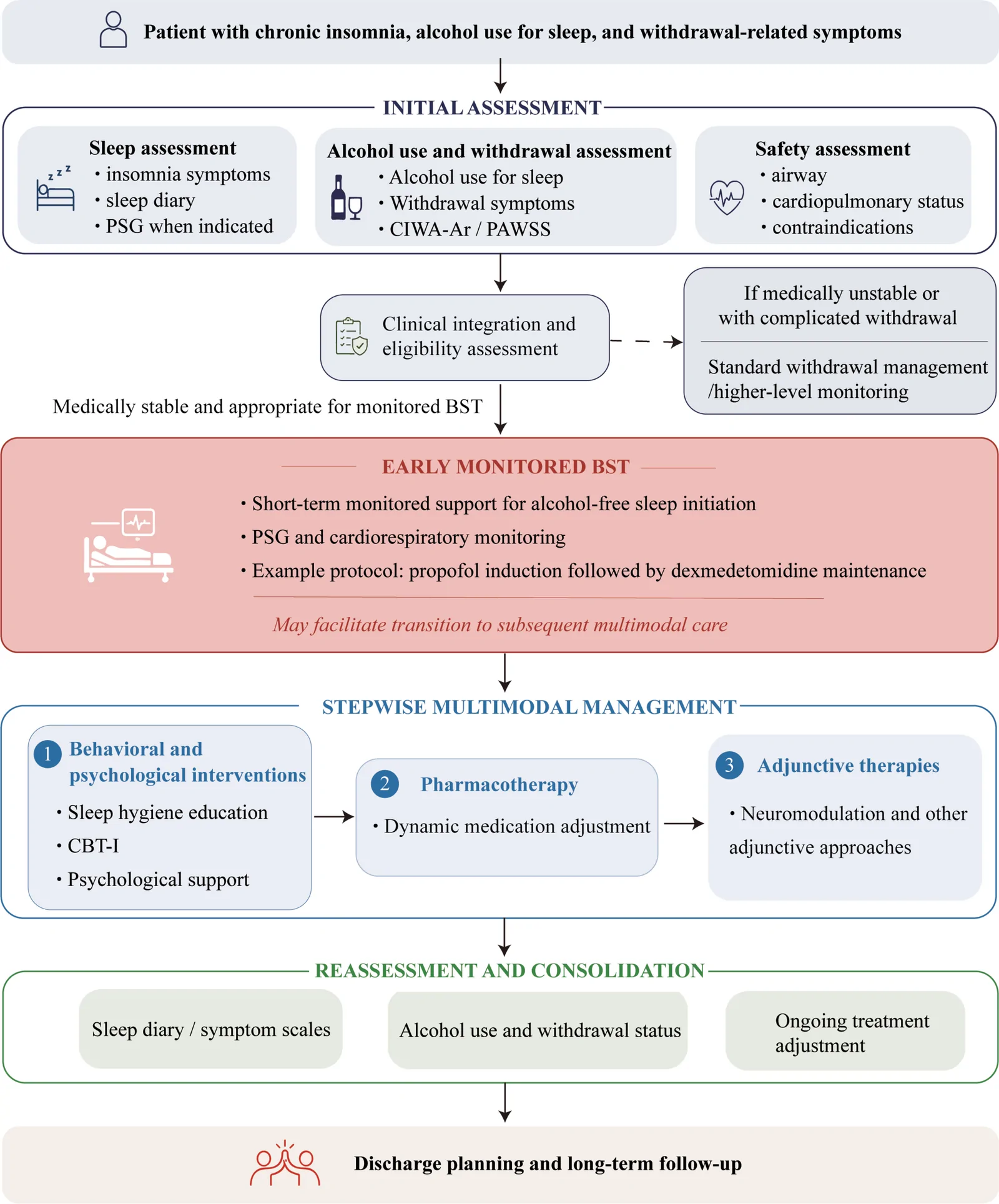
Case-derived conceptual framework for assessment and multimodal management of chronic insomnia with alcohol use for sleep and withdrawal-related symptoms. BST = bionic sleep therapy, CBT-I = cognitive behavioral therapy for insomnia, CIWA-Ar = Clinical Institute Withdrawal Assessment for Alcohol, Revised, PAWSS = Prediction of Alcohol Withdrawal Severity Scale, PSG = polysomnography.

### 3.1. The vicious cycle of insomnia, alcohol use for sleep, withdrawal-related insomnia, and resumption of drinking

The patient had long used alcohol to cope with difficulty initiating sleep, gradually developing a vicious cycle of “insomnia—alcohol use for sleep—withdrawal-related insomnia—resumption of drinking.”^[4]^ In the short term, alcohol enhances γ-aminobutyric acid type A (GABA_A_) receptor-mediated inhibitory neurotransmission,^[11]^ reduces arousal, and transiently facilitates sleep initiation.^[3]^ However, repeated long-term alcohol use induces neuroadaptive changes characterized by reduced sensitivity to its inhibitory effects, along with compensatory enhancement of glutamatergic excitatory neurotransmission,^[11]^ alterations in adenosine signaling,^[3]^ and dysregulation of stress-related systems.^[12]^ These alcohol-related neuroadaptations are accompanied by disturbances in sleep homeostasis and architecture.^[3]^ When alcohol intake is reduced or discontinued, its acute inhibitory effects dissipate while these neuroadaptations may persist, shifting central nervous system activity toward a hyperexcitable state and contributing to worsening insomnia, anxiety, irritability, and autonomic hyperactivity.^[8]^ Repeated use of alcohol to relieve insomnia and withdrawal-related discomfort may further reinforce the perception that alcohol is necessary for sleep, strengthening the entrenched belief that “sleep is impossible without alcohol” and perpetuating this cycle.^[4]^

In this case, the patient continued to rely on alcohol for sleep despite previous treatment with sleep medications, suggesting his sleep disturbance was influenced by neuroadaptation, withdrawal-related hyperarousal, and a long-established pattern of using alcohol as a sleep aid. In response, BST was introduced early and subsequently integrated with multimodal management. Under close monitoring, the first BST session was temporally associated with early improvement in withdrawal-related symptoms. During that night, the patient initiated sleep without alcohol and returned to sleep after a nocturnal awakening without further drinking. This experience provided an early opportunity to establish sleep without alcohol and proceed with subsequent cognitive and behavioral interventions. CBT-I, sleep hygiene education, psychological support, and medication adjustment were then reinforced to support continued sleep without alcohol and address sleep and withdrawal-related symptoms. The small amount of alcohol consumed on the night of the second BST session highlights the persistence of the patient’s long-established alcohol-for-sleep behavior. Nevertheless, no further alcohol intake was recorded during the remainder of hospitalization, while sleep initiation, maintenance, and several daytime symptoms gradually improved. Taken together, these observations suggest that, within multimodal management, BST may have contributed to the early improvement in difficulty initiating sleep without alcohol and may have facilitated transition to subsequent treatment; however, its specific contribution cannot be determined from this case.

### 3.2. Therapeutic strategy and potential value of BST

Propofol is a positive allosteric modulator of GABA_A_ receptors that enhances GABAergic inhibitory synaptic transmission, producing rapid sedative-hypnotic effects.^[13]^ Experimental studies show that propofol suppresses the activity of arousal-promoting glutamatergic neurons in the lateral hypothalamus and noradrenergic neurons in the locus coeruleus, attenuating output from arousal systems.^[14,15]^ Propofol-mediated unconsciousness is characterized by prominent slow-delta and α oscillations on electroencephalography.^[16]^ During alcohol withdrawal, reduced GABAergic inhibitory tone and relative excitatory predominance contribute to a hyperexcitable state.^[11]^ Clinically, this may manifest as insomnia, anxiety, agitation, and autonomic hyperactivity.^[8]^ Against this hyperexcitable state, the GABAergic and arousal-circuit effects of propofol provide a mechanistic rationale for its potential to attenuate withdrawal-related hyperarousal. Previous clinical evidence has also supported its adjunctive use in severe or refractory alcohol withdrawal.^[17]^ Its rapid onset, titratable depth of sedation, and relatively rapid recovery make it suitable for the induction phase of the present BST protocol and provide a pharmacologic basis for subsequent maintenance of a stable sleep-like state with dexmedetomidine.

Dexmedetomidine is a highly selective α2-adrenergic receptor agonist whose sedative effects are associated with reduced central noradrenergic activity. Animal studies further suggest that dexmedetomidine modulates sleep-related neural circuits, including those involving the ventrolateral preoptic area.^[18]^ During deeper levels of sedation, its electroencephalographic activity may show increased δ-band and slow-wave activity accompanied by spindle-like oscillations, producing a sleep-like sedative state that shares certain features with non-rapid eye movement sleep.^[19]^ However, pharmacologically induced sedation should not be regarded as equivalent to physiological sleep, and the absence of N3 sleep on PSG in the present case does not support an interpretation of normalized sleep architecture. During alcohol withdrawal, excessive noradrenergic and sympathetic activation is closely associated with anxiety, agitation, and autonomic hyperactivity. Dexmedetomidine has been evaluated as an adjunct to benzodiazepine-based therapy in alcohol withdrawal syndrome, particularly when persistent autonomic hyperactivity or anxiety is not adequately controlled.^[20]^ In addition, dexmedetomidine has relatively limited suppressive effects on respiratory drive, which may facilitate preservation of stable spontaneous ventilation under continuous cardiorespiratory monitoring.^[21]^ Accordingly, within the present BST protocol, dexmedetomidine primarily serves to maintain the sleep-like state and modulate sympathetic activity, complementing the rapid induction provided by propofol.

Taken together, propofol and dexmedetomidine may provide complementary effects in the present BST protocol in both temporal sequence and pharmacologic mechanism. Propofol has a rapid onset and produces prompt sedative-hypnotic effects, whereas dexmedetomidine provides ongoing modulation of noradrenergic and sympathetic activity to maintain a relatively stable sedative state. Previous clinical experience with the combined use of propofol and dexmedetomidine in severe alcohol withdrawal has been reported.^[22]^ In a study of deep sedation in older patients, adding dexmedetomidine to propofol was associated with reduced propofol requirements and fewer airway interventions, while also helping to maintain hemodynamic stability.^[23]^ These findings provide context for the potential dose-sparing effect of the combination and its monitored use in older patients. Accordingly, the present BST protocol employed a strategy of “rapid induction with propofol followed by maintenance sedation and sympathetic modulation with dexmedetomidine,” combining rapid induction with relatively stable maintenance of a sleep-like state and providing short-term, controllable pharmacologic support for withdrawal-related hyperarousal and difficulty initiating sleep. Similar central nervous system hyperexcitability and sympathetic overactivity may also occur during withdrawal associated with prolonged opioid or benzodiazepine exposure.^[24]^ These shared pathophysiologic features, together with preliminary evidence supporting α2-adrenergic modulation in opioid withdrawal,^[25]^ suggest that the pharmacologic rationale underlying BST may merit further investigation in other withdrawal states characterized by hyperarousal and autonomic overactivity.

## 4. Limitations

This case report has several limitations. First, this report describes only 1 older male patient, and no standard-treatment or single-agent control was available. Therefore, potential effects of natural fluctuations in clinical course and nonspecific treatment effects cannot be excluded, limiting the reproducibility and generalizability of the findings. Although propofol and dexmedetomidine have potentially complementary pharmacologic properties, this case cannot establish clinical synergy between the 2 agents or determine whether their combination provides additional benefit over either agent alone or conventional treatment. Second, because multiple interventions were implemented concurrently, the specific contributions of BST could not be distinguished from those of concomitant pharmacologic, behavioral, and other treatments. Accordingly, the observed changes cannot be attributed to BST alone, and this case does not establish its independent therapeutic effect. Third, outcome assessment was not sufficiently systematic. Objective sleep monitoring was limited, with no stable pretreatment baseline or repeated PSG assessments at multiple time points. Alcohol withdrawal-related symptoms and cravings were assessed primarily through clinical observation and patient self-report, and standardized instruments such as the Clinical Institute Withdrawal Assessment for Alcohol, Revised, were not administered serially during hospitalization. Consequently, dynamic changes in withdrawal severity could not be quantified objectively. Fourth, the patient received only 2 BST sessions and was observed for only 1 week during hospitalization, with no postdischarge follow-up data available. Therefore, the durability of observed effects and longer-term course of alcohol abstinence or relapse could not be assessed. Finally, this case documented only short-term tolerability of 2 monitored BST sessions and cannot address rare adverse events or long-term tolerability of repeated treatment.

Overall, this report provides only preliminary, short-term clinical observations. Prospective studies using standardized intervention protocols and structured follow-up are needed to further evaluate the therapeutic effects, tolerability, and durability of BST.

## 5. Conclusion

This case provides preliminary clinical observations suggesting that BST using propofol and dexmedetomidine combined with multimodal management may provide early transitional support and be associated with improved sleep initiation and continuity without alcohol in patients with long-term alcohol use as a sleep aid and withdrawal-related insomnia.

## Acknowledgments

The authors acknowledge the staff of the Sleep Therapy Clinical Medicine Center.

## Author contributions

**Conceptualization:** Qiu-Xia Xiao, Liu-Lin Xiong, Ruo-Lan Du.

**Data curation:** Shuai-Yu Zhu, Qiu-Xia Xiao, Zheng-Yuan Duan, Yan-Yan Ma, Peng-Cheng Zhao, Yi-Jun Bao, Jiang-Ming Lei, Lin Wang, Huan Zhou, Run-Xing Ma.

**Investigation:** Qiu-Xia Xiao, Zheng-Yuan Duan, Yan-Yan Ma, Peng-Cheng Zhao, Hong-Xia Chen, Yi-Jun Bao, Jiang-Ming Lei, Lin Wang, Huan Zhou, Run-Xing Ma, Hui-Li Pu.

**Methodology:** Qiu-Xia Xiao, Liu-Lin Xiong, Ruo-Lan Du.

**Supervision:** Kai-Feng Wu, Liu-Lin Xiong, Ruo-Lan Du.

**Writing – original draft:** Qiu-Xia Xiao, Zheng-Yuan Duan.

**Writing – review & editing:** Shuai-Yu Zhu, Qiu-Xia Xiao, Liu-Lin Xiong, Ruo-Lan Du.
